# Genome Sequence of an Alphaherpesvirus from a Beluga Whale (*Delphinapterus leucas*)

**DOI:** 10.1128/genomeA.01100-17

**Published:** 2017-10-19

**Authors:** Andrew J. Davison, Ole Nielsen, Kuttichantran Subramaniam, Jessica M. Jacob, Carlos H. Romero, Kathy A. Burek-Huntington, Thomas B. Waltzek

**Affiliations:** a MRC-University of Glasgow Centre for Virus Research, Glasgow, United Kingdom; b Department of Fisheries and Oceans Canada, Winnipeg, Manitoba, Canada; c Department of Infectious Diseases and Immunology, College of Veterinary Medicine, University of Florida, Gainesville, Florida, USA; d Alaska Veterinary Pathology, Eagle River, Alaska, USA

## Abstract

Beluga whale alphaherpesvirus 1 was isolated from a blowhole swab taken from a juvenile beluga whale. The genome is 144,144 bp in size and contains 86 putative genes. The virus groups phylogenetically with members of the genus *Varicellovirus* in subfamily *Alphaherpesvirinae* and is the first alphaherpesvirus sequenced from a marine mammal.

## GENOME ANNOUNCEMENT

A blowhole swab was obtained from a juvenile male beluga whale (DLBBN-AK_18-08_LN3131) that was sampled alive on 23 September 2008 as part of a health assessment project in Bristol Bay, Alaska (58° 46.304 N, 158° 45.794 W). The individual appeared unhealthy and lethargic and had numerous healed and active skin lesions. Cytopathic effects were observed within 1 month of inoculating this material onto primary beluga whale kidney (BWK) cells (1). Transmission electron microscopy revealed naked icosahedral nucleocapsids in various states of morphogenesis within the nuclei of infected cells, consistent with the presence of a herpesvirus. The virus was passaged three times in BWK cells before isolating DNA from the clarified medium. A sequencing library was prepared by using an Illumina Nextera XT DNA library preparation kit and analyzed by using a 600-cycle version 3 cartridge on an Illumina MiSeq instrument.

The data set of 7,188,388 quality-trimmed reads was assembled *de novo* by using SPAdes (2). A large herpesvirus-related contig was identified and joined to other contigs manually. The right genome terminus was identified from the presence of multiple reads commencing at the same position, and the adjacent nucleotide was assigned as the left terminus. Both termini exhibit sequence similarities to the genome termini of members of subfamily *Alphaherpesvirinae*. Problematic regions in heterogenous tandem reiterations were solved by adopting the longest solutions or by PCR. The linear viral genome (144,144 bp) consists of a long unique region (U_L_, 108,207 bp) flanked by an inverted repeat (TR_L_/IR_L_, 4,598 bp), and a short unique region (U_S_, 8,927 bp), also flanked by an inverted repeat (TR_S_/IR_S_, 8,907 bp), yielding the overall arrangement TR_L_-U_L_-IR_L_-IR_S_-U_S_-TR_S_, which is characteristic of members of subfamily *Alphaherpesvirinae*. The integrity of the sequence was assessed by inspecting a read assembly generated by using Bowtie 2 (3). A total of 489,292 reads (6.8% of the total) aligned at an average coverage of 719 reads per nucleotide.

A set of 86 genes was identified by an approach described previously (4). These genes include 11 that lack orthologues in other herpesviruses (Mo1 through Mo11), 6 that are duplicated in the inverted repeats (Mo1 through Mo4, RS1, and US1), one that is partially deleted (UL4), 2 versions of UL47, and 4 versions of RL2. Phylogenetic analyses showed that the virus groups with members of genus *Varicellovirus*, subfamily *Alphaherpesvirinae*.

Cases of herpesvirus infection in beluga whales have been reported previously in association with chronic dermatitis in a female (5) and a papilloma-like penile lesion in a male (6). Also, a systemic herpesvirus infection was determined to be the cause of death in a juvenile beluga whale, although only routine histopathology and electron microscopy were used in the diagnosis (7). Short sequences obtained from the penile lesion (6), and several other short sequences obtained from various beluga whale samples, are related very closely to the corresponding regions in the sequenced genome.

### Accession number(s).

The genome sequence of beluga whale alphaherpesvirus 1 strain LN3131-1 (proposed species *Monodontid alphaherpesvirus 1*) has been deposited in GenBank (accession no. MF678601).
